# Increased CD8 Tumor Infiltrating Lymphocytes in Colorectal Cancer Microenvironment Supports an Adaptive Immune Resistance Mechanism of *PD-L1* Expression

**DOI:** 10.31557/APJCP.2019.20.11.3421

**Published:** 2019

**Authors:** Aru W Sudoyo, Antonius N Kurniawan, Gita D Kusumo, Kusumo Kusumo, Fritzie A Rexana, Muhammad Yunus, Akterono D Budiyati, Dicky Kurniawan, Andi Utama, Ahmad R Utomo

**Affiliations:** 1 *Division of Hematology and Medical Oncology, Department of Internal Medicine, Universitas Indonesia, *; 2 *Medistra Hospital, *; 3 *Stem Cell and Cancer Institute, Jakarta, Indonesia. *

**Keywords:** CD8, Colorectal Cancer, PD-L1

## Abstract

**Background:**

Tumor cells express programmed death ligand-1* (PD-L1)* through several biological processes, thereby having different clinical significance depending on the underlying mechanism of expression. Currently, mechanisms leading to *PDL1* gene expression in colorectal cancer (CRC) are not fully understood.

**Methods:**

We investigated 98 Indonesia CRC patients to determine *PD-L1* protein expressions and their correlations with *PD-L1* gene copy number status, tumor infiltrating lymphocytes (TILs), tumor mutational profile, as well as clinicopathologic features.

**Results:**

Our investigation demonstrated that 18% of patients positively expressed *PD-L1*. Further analysis on *PD-L1* copy number revealed that all *PD-L1*^+^ tumors had normal copy number, indicating that the expression of *PD-L1* was not a consequence of genetic amplification of *PD-L1*. From TILs analysis, there was a significant increase of CD8 in all tumor cells expressing *PD-L1* (P=0.0051), indicating that the inducible *PD-L1* expression was the prominent mechanism occurred in CRC. Furthermore, the expression of *PD-L1* in this CRC population was significantly associated with high frequency of MSI compared to the remainder *PD-L1*^- ^tumors (P=0.0001), suggesting the natural immunogenicity of tumors via MSI status plays role in attracting immune response. On the other hand, p53 mutations which were frequently observed within Indonesian CRCs (76.5%), they were not associated with *PD-L1* expression (p=0.1108), as well as *KRAS *gene (29.6%; p=0.5772) and *BRAF* gene mutations (5%; p=0.2171).

**Conclusion:**

Our study demonstrated that *PD-L1* expressions in CRC were predominantly found as a consequence of infiltrating CD8 T lymphocytes that in part arise in the setting of microsatellite instability. Taken together, our findings further support the role of adaptive immune resistance to drive *PD-L1* induction in tumor microenvironment and may provide important rationale for strategy implementation of immunotherapy for CRC cases.

## Introduction

Colorectal cancer is an emerging health problem in Indonesia, and currently ranks among four highest cancer mortality rate with 16,034 cases per year or 8.5% of total cancer-related mortality in Indonesia (Bray et al., 2018). Most cases are found in late or metastatic stage. Lack of awareness on early detection in high risk population and western lifestyle are suspected to contribute to this situation (Abdullah et al., 2012). For most advanced/metastatic CRC patients, chemotherapy using cytostatics is the only viable option, with a 5-year survival between 5 and 70 percent depending on the stage and response. Newer cytostatics have not extended survival significantly and the so-called “targeted therapy” yields modest survival benefit (Venook and Saltz, 2013). Current understanding of immune-oncology has raised excitement in targeting *PD-1*/*PD-L1* as a promising option for treating advanced/metastatic CRC expressing *PD-L1* and/or harbouring microsatellite instability (MSI) (Birendra et al., 2017; Spallanzani et al., 2018). Nevertheless, considering that CRC is a heterogeneous disease and many factors contribute to *PD-L1* expression, we intended to determine which regulatory mechanisms play significant roles in *PD-L1* expression that is indispensable prerequisite of immunotherapy. 

Programmed death ligand-1 (*PD-L1*) is an immune inhibitory molecule that supresses the activation of T-cell upon binding to its receptor, *PD-1* (Pardoll, 2012; Kythreotou et al., 2018). In normal cells, this mechanism aims to maintain immune homeostasis. In contrast, the same mechanism has been exploited by tumor cells to evade or block host immune surveillance. Currently, inhibition of *PD-1*/*PD-L1* interaction to restore anti-tumor immunity has shown a remarkably durable clinical response rate in patients, notably in melanoma, renal, lung, prostate and bladder carcinoma (Llosa et al., 2015). 

Expression of *PD-L1* is, in general, associated with response to anti *PD-1*/*PD-L1* monoclonal antibody. However, the expression of *PD-L1* is regulated in different ways, which may affect different and significant responses to therapy. Two mechanisms of *PD-L1* expressions have been shown previously. First, constitutive *PD-L1* expression on tumor cells are determined by cancer-driving gene alteration. Second, *PD-L1* expression is inducible as a response to host adaptive immune resistance (Ribas and Hu-Lieskovan, 2016; Shi, 2018). In this study, we aimed to investigate the tendency of *PD-L1* expression mechanism in CRC patients by determining its correlation with *PD-L1* gene copy number status, tumour infiltrating lymphocytes (TILs), tumor mutational profile, as well as clinicopathologic features.

## Materials and Methods

*Sample collection and slide preparation*

Ninety eight formalin fixed paraffin embedded (FFPE) tumor tissue blocks from 98 CRC patients along with paired haematoxylin-eosin (HE) stained tissue slides were obtained from Medistra Hospital upon approval by Medistra ethic committee. All blocks were sectioned into 4 µm thin specimens and mounted on coated sample slides for further assessment which were conducted in Stem Cell and Cancer Institute (SCI) laboratory.

*Isolation of genomic DNA from FFPE tissue*

Tumor enriched areas were marked by senior pathologist on HE slides and corresponding areas from the unstained slide were manually scrapped using sterile needle. The paraffin flakes were then transferred into 1.5 ml tube, deparaffinised with 1 ml of xylene, vortexed and centrifuged to pellet the tissue. The tissue pellet was then washed with 70% alcohol twice and then proceeded to DNA extraction step. We used QIAamp DNA FFPE Tissue Kit (Qiagen, Germany) and followed the instructions as described in kit protocol. Final DNA was diluted in 20 µl nuclease free water (Qiagen, Germany) and stored at -20^o^C until further used.

*PD-L1 Copy number assays*

Copy number analyses of *PD-L1* were performed on 18 positive tumor tissue specimens using TaqMan copy number assay kit specific for *PD-L1* and RNAse P, (Hs01477451_cn, Applied Biosystems) on a StepOnePlus Real Time PCR System (Life Technologies) according to manufacturers’ instructions and described elsewhere (Ikeda et al., 2016). Briefly, polymerase chain reaction (PCR) was performed with TaqMan Genotyping Master Mix (Life Technologies). Each single-well reaction contained 20 ng of genomic DNA and was run for *PD-L1* gene (FAM dye-labeled probe) and *Rnase P* gene (VIC dye-labeled probe) simultaneously. Copy number were then calculated with CopyCaller v2.0 Software (Life Technologies) using ΔΔ Ct relative quantification method. The results were then calculated as relative copy number of *PD-L1* gene normalized to RNAse P. Blood samples from healthy subjects were used as reference. A *PD-L1* copy number of 3 or greater was defined as amplification positive, whereas a *PD-L1* gene copy number less than 3 was defined as negative.

*PD-L1 expression, evaluation of TILs and immunoscore calculation*

Immunohistochemistry (IHC) was performed using the following antibodies: Rabbit *anti-PD-L1* XP^®^ mAb at dilution of 1:300 (EIL3N), Rabbit anti-CD3-epsilon at dilution of 1:300 (D7A6E) XP^®^ mAb, and Rabbit anti-CD8-alpha at dilution of 1:300 (D8A8Y) mAb (all were manufactured by Cell Signaling Technology, USA). Briefly, sections were deparaffinized with serial xylene dipping followed by an ethanol gradient for rehydration. Sections were then incubated in 3% hydrogen peroxide to block endogenous peroxidase activity followed with antigen retrieval using EDTA buffer. After washing with Tris Buffered Saline with Tween^®^ 20, sections were then incubated overnight at 4^o^C with primary antibody. The expression of *PD-L1*, CD3 and C8 were detected using ChemMate EnVision Detection Kit with DAB substrate (Dako, USA) following the manufacturer’s instruction. Panoramic Scanner (3dHistech, Hungary) was then used for image acquisition and processing. 

*PD-L1* positivity was defined as *PD-L1* expression on ≥ 5% of membranous positive cell staining of any intensity. Immunoscore (IS) assessment was carried out based on density of each CD3^+^ and CD8^+^ TILs in two tumor areas, namely centre of tumor (CT) and invasive margin (IM) using digital pathology software (QuPath, UK; Bankhead et al., 2017). TIL densities were further being classified as high (valued as 1) or low (valued as 0) according to the density cut off (Anitei et al., 2014) and summed up to generate numerical IS scales ranging from 0-4.

We used anti-human *PD-L1* antibody clone E1L3N^®^ from Cell Signalling Technology (USA), in ratio of 1:300 with overnight incubation time. Rabbit XP^®^ was then used as secondary antibody followed by counterstaining with haematoxylin and slides dehydration. Expression of *PD-L1* by cells*PD-L1* expression was identified based on stained cell within the cytoplasm or on the cell surface. *PD-L1* positivity was determined by using the 5% cut off. Tumor infiltrating lymphocytes (TIL) intensity was assessed by immunohistochemistry assay. We used CD3-epsilon (D7A6E) XP (R) Rabbit mAb) and CD8-alpha (D8A8Y) Rabbit mAb (Cell Signaling Technology, USA). The antibody ratio and incubation time was equal to previous *PD-L1* staining process. Stained slides for *PD-L1* and TILs were then scanned using Panoramic Scanner (3dHistech, Hungary) for digital imaging process. Only TILs slide that was further analyzed using digital pathology software (QuPath, UK; Bankhead et al., 2017) for calculation of positive cells densityto calculate density of stained cells. Immunoscore (IS) was determined based on density of each CD3^+^ and CD8^+^ TILs in two tumor areas, namely centre of tumor (CT) and invasive margin (IM). TIL densities were further being classified as high (valued as 1) or low (valued as 0) according to the density cut off (Anitei et al., 2014) and summed up to generate numerical the IS scales ranging from 0-4 which comprised of IS 0 to IS 4.

**Table 1:** Summary of Clinicopathological and Molecular Profile of CRC Subset

	All (n=98)		PD-L1 Expression		
	n	%	Positive (n=18)	Negative (n=80)	p-Value (p<0.05)
Gender					
Male	56	57.1%	10	46	1.000
Female	42	42.9%	8	34	
Age					
Median	69		-	-	
Range	10-93		-	-	
Unknown	4		-	-	
Metastases					
Lung	13	13.3%	-	-	
Liver	20	20.4%	-	-	
Lymph Node	15	15.3%	-	-	
Others^†^	6	6.1%	-	-	
None^1^	27	27.6%	-	-	
Unknown^2^	24	24.5%	-	-	
Differentiation					
Well or Moderate	79	80.6%	14	65	0.3148
Poor or Mucinous	6	6.1%	2	4	
Unknown	13	13.3%	2	11	
Location					
Left	67	68.4%	8	59	0.0190*
Right^3^	21	21.4%	8	13	
Unknown^2^	10	10.2%	2	8	
Microsatellite					
MSS	88	89.8%	10	78	0.0001*
MSI	10	10.2%	8	2	
Biomarker					
*KRAS WT*	68	69.4%	14	54	0.5722
*KRAS Mut*	29	29.6%	4	25	
Unknown^2^				1	
*BRAF WT*	90	91.8%	15	75	0.2171
*BRAF Mut*	5	5.1%	2	3	
Unknown^2^			1	2	
p53 WT	18	18.4%	6	12	0.1108
p53 Mut	75	76.5%	12	63	
Unknown^2^				5	

Others include Brain, Ovarian, Kidney, Bones and Pancreas; No metastases found; No data due to incomplete information, insufficient sample or inconclusive result; Include, ascending colon- cecum and ileum colon; * Indicating significance level (p) < 0.05; PD-L1, Programmed death-ligand 1 (PD-L1); MSS, microsatellite stable; MSI, microsatellite instability; WT, wild type; Mut, mutation.

**Figure 1:**
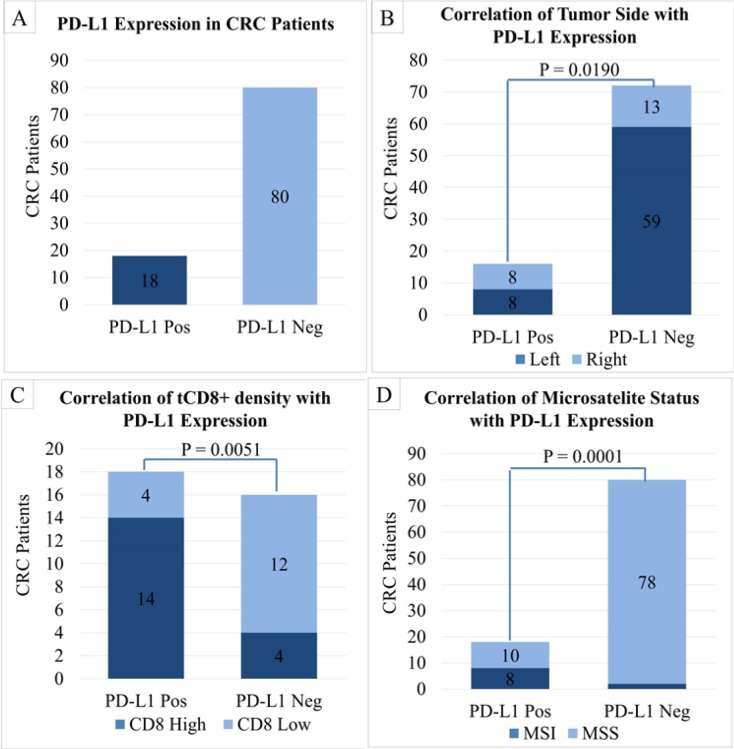
Graphs of Correlation between *PD-L1* Status and other Biomarkers. A). Graph showing *PD-L1* profile among CRC patients, B). Graph showing a significant correlation between *PD-L1* status and tumor side, C). Graph showing correlation between tCD8+ in CT with *PD-L1* status, D). Graph showing correlation between MSI status with *PD-L1* Status

*Microsatellite status and p53 mutation analysis*

Microsatellite and *p53* mutation status were determined by IHC as described above. Microsatellite status were determined using Rabbit monoclonal antibodies against four mismatch repair proteins (MMR); MLH1 (ES05), MSH2 (FE11), MSH6 (EP49), and PMS2 (EP51) (all were manufactured by Agilent, USA). Each antibody was used at ratio of 1:100 prior to primary antibody incubation for one hour. Adjacent normal tissue served as an internal control for positive staining and a negative control staining was carried out without the primary antibody. Specimens with deficient nuclear expression of MMR proteins in the tumour cells but proficient in normal cells were identified as microsatellite instable (MSI). 

The presence of *p53* mutation was detected using monoclonal antibody against *p53* (DO-7, Agilent, USA) at ratio of 1:500 prior to primary antibody incubation for one hour. Specimens displayed restricted overexpression pattern were identified as *p53* wild type, while specimens showed diffused or complete negative expression were identified as mutated *p53* (Nyiraneza et. al., 2012). 

*KRAS and BRAF genotyping*

Mutations on *KRAS *and* BRAF* genes were assessed using high resolution melting (HRM) method. The protocols from previous publications were adopted for the mutation analysis of *KRAS *(Levi et al., 2017) and *BRAF* (V600E) (Kristensen et al., 2011). Assessment using ABI 3500 Genetic Analyzer was performed for further validation of *KRAS *mutation in samples that exhibited split peak pattern of PCR product melt-curves. 

*Statistical Analysis*

The Fisher-Exact test for categorical variables was used to determine the significance between clinicopathological and molecular data. P<0.05 was considered statistically significant.

## Results

*The characteristics of CRC patients*

This study investigated 98 CRC specimens that were collected from 2010 – 2017. The median age of patients was 69, ranging from 10 to 93 years old, whereas 57.1% of them were females. About 55.1% (54/98) had metastasis to distant organs, and histologically, 80.6% were well or moderately differentiated. Most tumours were located at the left side of the colon (68.4%; 67/98). Table 1 described the clinicopathological and molecular characteristics of patients stratified by *PD-L1* status. 

*PD-L1 expression and copy number variation*

We began our investigation on *PD-L1* expression profiles. As seen in Table 1, we identified 18.37% (18/98) of patients expressed *PD-L1* in their tumours (Figure 1.A). These *PD-L1*^+ ^positive tumours consisted of equal percentage between the left-sided and the right-sided CRC cases (50.0%; 8/16 for each side). On the other hand, *PD-L1*^-^ negative tumours were found more often on left side of colon (81.9%; 59/72; P=0.0190) since more than half of Indonesian CRC population suffered from left-sided CRC (Figure 1.B). No further significant of difference was observed between these two subsets based on age and gender, as well as grade of tumour differentiation.

In order to confirm whether *PD-L1* amplification contributes to *PD-L1* expression, analysis on *PD-L1* gene copy number was then performed. In the present study, we used DNA isolated from healthy blood donor as our control. None of our CRC’s *PD-L1* DNA copy number exceeded more than two copies. Our result showed that *PD-L1* amplification was not commonly occurred in CRC population.

*Patterns of tumor infiltrating lymphocytes (TIL), CD8* ^+^ *density and correlation with PD-L1 expression*

Our immunostaining analysis demonstrated that TILs were present in both *PD-L1*^+^ and *PD-L1*^-^ tumor samples (Supplement and Supporting Data/SSD 1). From 18 positive *PD-L1* specimens, 9 specimens were examined for immunoscore (IS) value due to the availability of centre of tumor (CT) and invasive margin (IM) areas. Majority of *PD-L1*^+^ tumors (55.6%) had high IS of 4 in their tumor microenvironment (5/9), while the other 11.1% of patients had IS 3 (1/9) and 33.3% had IS 2 (3/9). On the other hand, IS 3 was frequently found in our *PD-L1*^-^ subset (57.1%; 8/14), followed by IS 2 (28.6%; 4/14) and IS 4 (14.3%; 2/14). 

Regarding the subset of TILs, further analysis on CD8^+^ density in centre of tumor area (tCD8^+^) demonstrated that (77.7%;14/18) *PD-L1*^+^ tumors contained a high number of tCD8^+^. On the other hand, representative samples of *PD-L1*^-^ subset of patients (25.0%; 4/16) demonstrated a significantly lower tCD8^+^ density (Figure 1C) (P=0,0051). 

*Microsatellite instability and mutational status*

Table 1 summarized several molecular characteristics, that known to contribute to CRC development, stratified by *PD-L1* status. We found that 89.8% (88/98) of CRC patients were determined to be microsatellite stable (MSS) while 10.2% (10/98) were MSI. There was a significant association between *PD-L1* expression with MSI frequency, as shown by 80% (8/10) of MSI tumor also expressed *PD-L1* compared to MSS tumors which only 11.3% (10/88) expressed *PD-L1* (Figure.1D) (P= 0.0001). 

Based on our *p53* immunostaining, 76.5% (75/98) of patients harboured mutation on their *p53*. Regarding to *PD-L1* status, 12 of 18 *PD-L1*^+^ cases (66.7%) had *p53* mutation while the rest (6/18) remains wild type. In addition to microsatellite status, MSS subset of patients tended to harbour *p53* mutation (83.3%; 70/84) compared to MSI patients (55.6%; 5/9; P=0.0671).

Meanwhile, we found 29.6% (29/98) of CRC patients harboured *KRAS *mutations, and 5.1% (5/98) had *BRAF* mutation V600E. Neither *KRAS *nor *BRAF* mutational status were associated with *PD-L1* expression. 

Overall, based on molecular characteristics, 82 of our CRC patients exhibited one or more mutations, with 59 harbouring one mutation and 23 harbouring two mutations (SSD 2). These included 8 (8.2%) with *KRAS/BRAF *gene mutations, 50 (51.0%) with *p53* mutations and 23 (23.5%) with *KRAS/BRAF *in addition to *p53* mutations. 

## Discussion

*PD-L1* is expressed on tumour cells in many type of malignancies and implies a weakened host immune response and consequent poor prognosis (Anitei et al., 2014). With regard to CRC, *PD-L1* expression is found in small subset of patients, ranging from 9% to 15% which is similar with our result (18.4%) (Rosenbaum et al., 2016; Valentini et al., 2018). To date, two mechanisms underlying this upregulation of *PD-L1* have been reported (Ribas and Hu-Lieskovan, 2016; Shi, 2018). Firstly, amplification in *PD-L1* locus is correlated significantly to constitutive *PD-L1* expression in malignant cells (Ikeda et al., 2016; Ribas and Hu-Lieskovan, 2016). Secondly, Inducible *PD-L1* expression mechanism refers to adaptive immune resistance in response to local inflammatory signals (e.g. IFN-γ) which are produced by active anti-tumor immune response (cytotoxic T-cell and/or Th1 pathway activation) (Sanmamed and Chen, 2014).

One of the prominent factors in inducing *PD-L1* expression is the interferon-γ (IFN-γ) acting mainly via the JAK/STAT1/interferon regulatory factor (IRF) 1 pathway in multiple types of cancers (Ikeda et al., 2016; Moon et al., 2017). As a consequence of immune response to tumor antigens, attracted cytotoxic T-cells produce IFN-γ within tumor microenvironment, which further leads to the expression of *PD-L1* by any surrounding cells (Tumeh et al., 2014; McDermott et al., 2016). CD8^+^ T cells are cytotoxic T lymphocytes that directly attack cancer cells and play a central role in anti-cancer immunity. Previous study shows substantial evidence that the density of CD8^+^ TILs was associated with the long-term survival in patients with various types of cancer (Anraku et al., 2008; Yao et al., 2017; Eriksen et al., 2018). In CRC tissue, *PD-L1* expression is inversely associated with FoxP3^+^, but not CD3^+^, CD8^+^ or CD45RO^+^ cell density (Kim et al., 2017). According to this, we analysed TIL density based on CD3^+^ and CD8^+^ presence using immune scoring technique developed by Galon et al., (2014). Our result demonstrated a strong association between upregulation of *PD-L1* and high density of CD8^+^ within centre of tumor region (Figure 1C). The data likewise clearly explained why *PD-L1*^-^ subset had lower immunoscoring level compare to *PD-L1*^+^ subset. 

Our analysis on CD8^+^ density in centre of tumor area (tCD8^+^) demonstrated that 14 (77.8%) *PD-L1*^+^ tumors contained a high number of tCD8^+^ (cut off value 202) (Anitei et al., 2014). The presence of tumor infiltrating lymphocytes (TILs) may induce *PD-L1* expression through binding of IFN-γ produced by CD8^+^ T cells to receptor on tumor surface. This ligand-receptor binding thus activates downstream pathway to *PD-L1* expression (Garcia-Diaz et al., 2017).

Four types of TIL and *PD-L1* expression status have been proposed to predict tumour responses to immune checkpoint inhibitors: type I (*PD-L1*^+^/*TIL*^+^; adaptive immune resistance), type II (*PD-L1*^-^/*TIL*^-^; immunological ignorance), type III (*PD-L1*^+^*/ TIL*^-^; intrinsic induction) and type IV (*PD-L1*^-^*/TIL*^+^; tolerance) (Teng et al., 2015). Based on these classifications we could presume that adaptive immune resistance was predominantly found as the mechanism underlying the *PD-L1* expression in CRC. To our knowledge, there is no published data exist regarding the predictive value of *PD-L1* expression and TILs for anti *PD-1*/*PD-L1* therapy so far, however similar mechanism was found in breast cancer and CRC as well (Li et al., 2016; Kitano et al., 2017).

The majority of CRCs developed via a chromosomal instability pathway, and approximately 12-15% have deficiency in the mismatch repair (MMR) gene which is responsible for MSI (Ahn et al., 2016). The defect in MMR gene facilitates production of aberrant proteins acting as neo-antigens burdens (Xiao and Freeman, 2015). Some of these neo-antigens will be processed, presented on MHC, and recognized as foreign by T-cells thus attracting immune response within tumor microenvironment. In our small retrospective study, which showed small percentage of MSI patients (10.2%), there was a significant correlation between *PD-L1* upregulation and MSI status (P=0.0001). This intimate association between MSI and *PD-L1* expression has also been reviewed previously (Gatalica 2016). Presumably, this high neo-antigen burden might be one explanation for the high level of TIL-related induced *PD-L1* expression in CRC as described previously (Hodges et al., 2017; Yi et al., 2018). Nevertheless, adaptive immune resistance caused by tumor’s MSI status was not the main factor in inducing the expression of *PD-L1* as more than half of our *PD-L1*^+^ tumors were MSS. Previous study by Llosa et al., (2015) reported similar result using cell lines model. They explored *PD-L1* expression in MSI and MSS CRC cell lines and found that in response to IFN-γ both cell lines were modestly upregulated *PD-L1* and HLA-DR (Cooks et al., 2013).

One of proposed mechanism might be used to explain the mechanism of *PD-L1* expression in MSS tumors is the role of NF-κB. The expression of *PD-L1* in cancer cells is dependent on transcription factor NF-κB. There are NF-κB binding sites in the promoter region of the *PD-L1* gene (Shi, 2018). In addition, a study conducted by Cooks et al., (2013) demonstrated that *p53* mutation prolonged NF-κB activation and promoted chronic inflammation towards CRC as described previously (Galon et al., 2014). Our previous study on molecular profiling of Indonesian CRC patients demonstrated high frequency of NF-κB activation (73.5%) (Abdullah et al., 2012). This finding was in concordance with high frequency of *p53* mutation found in our population, indicating chronic inflammatory pathway might be maintained in majority of population by the absence of *p53* in tumor. With regard to our *PD-L1*^+^/*MSS* tumors, although it did not reach significant association, *p53* mutation was predominantly found within this subset. Presumably, we could speculate that TIL presence within tumor microenvironment was arise in the setting of chronic inflammation pathway.

Recent advances in CRC immunotherapy suggest MSI tumors will benefit from checkpoint inhibitors therapy (Birendra et al., 2017; Spallanzani et al., 2018). As reported in CRC phase II clinical trials setting, by Lee et al., (2015) after the administration of Pembrolizumab (an anti *PD-L1* mAb), a partial objective response rate of 40% were observed in MSI compare to 0% MSS CRC patients. Moreover, a CRC phase II trial by Overman et al., (2017) highlighted a partial response to Nivolumab alone or in association with Ipilimumab in 31% of MSI patients versus 10% of MSS patients. 

In addition to RAS pathway, although other study reported there were correlations between *PD-L1* expression with *BRAF* mutation in CRC (Rosenbaum et al., 2016), so far we could not find any relationship of RAS/*BRAF* mutation with *PD-L1* expression. 

Regarding to our clinicopathological data, although it did not reach significant, tumors that did not express *PD-L1* were most likely left-sided CRC (81.9%), whereas tumors expressing *PD-L1* shared equal proportion (50.0%) (P=0.0190) (Figure 1B). There is evidence of different response to treatment regarding tumor localization in CRC patients (Ulivi et al., 2017). Interesting result was found when we stratified tumor location based on *PD-L1* expression and then correlated it with microsatellite status. Our study showed that *PD-L1*^+^/*MSI* subset was found mostly at the right side of colon (71.4%), which was commonly found in CRC and it was predicted to have more favourable prognosis compare to other types of CRCs (Sugai et al., 2006; Ulivi et al., 2017; Valentini et al., 2018).

In conclusion, our study demonstrated that *PD-L1* expression in CRC was predominantly found as a consequence of infiltrating CD8 T lymphocytes that in part arise in the setting of microsatellite instability and high neo-antigen load, or in the setting of chronic inflammation pathway. This finding supports the role of adaptive immune resistance to drive *PD-L1* induction in tumor microenvironment and may provide important rationale for strategy implementation of immunotherapy for CRC.

## Supplement and Supporting Data (SSD)

SSD 1. Figure CD3 and CD8 presence in various IS within PD-L1 positive and negative subset

SSD 2. Table Mutation Burden in CRC Subset Patients

SSD 3. Quantitative PCR-based DNA copy number analysis of PD-L1 in 18 positive samples

SSD 4. Gel showing qPCR products of DNA copy number analysis of PD-L1 from 18 positive samples
